# Obituary

**Published:** 1887-08

**Authors:** Mark Hopkins


### Obituary.

MARK HOPKINS, M.D., L.L.D.

It may not be generally known that the Rev. Dr. Hopkins, President of Williams College, who died last month, was a doctor of medicine. But such is the case. Mark Hopkins graduated in medicine, in New York, in 1829, and made an attempt to enter into practice by forming a partnership with Dr. Silas West, of Binghampton, N. Y. At that time Dr. West could not acceed to the wish of Dr. Hopkins, and three months later the young physician was called to the Professorship of Rhetoric and Moral Philosophy in Williams College. This event diverted him from the practice of his profession and led him into the career which has been so honorable to himself and so useful to Williams College and to the world. Probably no college president has ever enjoyed more of the love and respect of his students than did President Hopkins, whose example and teachings were of the noblest sort. And, although he was but a short time in the ranks of the active members of the medical profession, it is a pleasure to believe that some share of his success may have been due to the ideas received during the period of his medical studies and medical work.

